# Phenotypic profiling of CD8^+^ T cells during *Plasmodium vivax* blood-stage infection

**DOI:** 10.1186/s12879-015-0762-x

**Published:** 2015-01-31

**Authors:** Natália Satchiko Hojo-Souza, Dhelio Batista Pereira, Lívia Silva Araújo Passos, Pedro Henrique Gazzinelli-Guimarães, Mariana Santos Cardoso, Mauro Shugiro Tada, Graziela Maria Zanini, Daniella Castanheira Bartholomeu, Ricardo Toshio Fujiwara, Lilian Lacerda Bueno

**Affiliations:** Departamento de Parasitologia, Instituto de Ciências Biológicas, Universidade Federal de Minas Gerais, Av. Antônio Carlos 6627, 31270-901 Belo Horizonte, Minas Gerais Brazil; Centro de Pesquisa em Medicina Tropical, Porto Velho, Rondônia Brazil; Instituto de Pesquisa Clínica Evandro Chagas, Fundação Oswaldo Cruz, Rio de Janeiro, Rio de Janeiro Brazil

**Keywords:** *Plasmodium vivax*, Malaria, CD8^+^ T lymphocytes

## Abstract

**Background:**

For a long time, the role of CD8^+^ T cells in blood-stage malaria was not considered important because erythrocytes do not express major histocompatibility complex (MHC) class I proteins. While recent evidences suggest that CD8^+^ T cells may play an important role during the erythrocytic phase of infection by eliminating parasites, CD8^+^ T cells might also contribute to modulate the host response through production of regulatory cytokines. Thus, the role of CD8^+^ T cells during blood-stage malaria is unclear. Here, we report the phenotypic profiling of CD8^+^ T cells subsets from patients with uncomplicated symptomatic *P. vivax* malaria.

**Methods:**

Blood samples were collected from 20 *Plasmodium vivax*-infected individuals and 12 healthy individuals. Immunophenotyping was conducted by flow cytometry. Plasma levels of IFN-γ, TNF-α and IL-10 were determined by ELISA/CBA. Unpaired t-test or Mann–Whitney test was used depending on the data distribution.

**Results:**

*P. vivax*-infected subjects had lower percentages and absolute numbers of CD8^+^CD45RA^+^ and CD8^+^CD45RO^+^ T cells when compared to uninfected individuals (p ≤ 0.0002). A significantly lower absolute number of circulating CD8^+^CD45^+^CCR7^+^ cells (p = 0.002) was observed in *P. vivax*-infected individuals indicating that infection reduces the number of central memory T cells. Cytokine expression was significantly reduced in the naïve T cells from infected individuals compared with negative controls, as shown by lower numbers of IFN-γ^+^ (p = 0.001), TNF-α^+^ (p < 0.0001) and IL-10^+^ (p < 0.0001) CD8^+^ T cells. Despite the reduction in the number of CD8^+^ memory T cells producing IFN-γ (p < 0.0001), *P. vivax*-infected individuals demonstrated a significant increase in memory CD8^+^TNF-α^+^ (p = 0.016) and CD8^+^IL-10^+^ (p = 0.004) cells. Positive correlations were observed between absolute numbers of CD8^+^IL-10^+^ and numbers of CD8^+^IFN-γ^+^ (p < 0.001) and CD8^+^TNF-α^+^ T cells (p ≤ 0.0001). Finally, an increase in the plasma levels of TNF-α (p = 0.017) and IL-10 (p = 0.006) and a decrease in the IFN-γ plasma level (p <0.0001) were observed in the *P. vivax*-infected individuals.

**Conclusions:**

*P. vivax* infection reduces the numbers of different subsets of CD8^+^ T cells, particularly the memory cells, during blood-stage of infection and enhances the number of CD8^+^ memory T cells expressing IL-10, which positively correlates with the number of cells expressing TNF-α and IFN-γ.

**Electronic supplementary material:**

The online version of this article (doi:10.1186/s12879-015-0762-x) contains supplementary material, which is available to authorized users.

## Background

Human malaria is caused by protozoa that belong to the genus *Plasmodium*, mainly *P. falciparum* and *P. vivax*. In Brazil, the Amazon region is endemic for malaria, and there is a high prevalence of *P. vivax* malaria (85% of cases), which has elevated the morbidity rate [1]. For malaria, naturally acquired protective immunity (lower risk of disease/lower parasitemia/asymptomatic disease) can be achieved only after repeated infections [2] and does not confer sterile immunity. For example, even though naturally acquired immunity protects against symptomatic malaria, a recent study on individuals living in the Mali endemic area found no evidence of acquired sterile immunity to *P. falciparum* infection [3].

B cells and CD4^+^ T lymphocytes play an important protective role during the blood stage of malaria infection [4], and CD8^+^ T cells play a critical role in pre-erythrocytic immunity. Studies using experimental models have shown that these cells directly promote the lysis of infected hepatocytes and parasite death, and these events are mediated by IFN-γ, perforin and granzyme B [5]. For a long time, the role of CD8^+^ T cells in the blood stage of malaria was considered minor because erythrocytes do not express major histocompatibility complex (MHC) class I proteins [6,7]. Very few studies focusing on the function of CD8^+^ T cells during blood-stage infection have been reported because there is some agreement among researchers that these cells only play an important role in the liver-stage of malaria. However, recent studies have suggested that CD8^+^ T cells may play a role in eliminating parasites during the blood stage of infection [8,9]. An increase in the number of effector memory CD8^+^ T cells in response to infection with lethal *P. yoelli* was observed in recipient mice that received CD8^+^ T cells from immune mice [8]. Using animals genetically deficient for PD-1 (a molecule with particular importance in cell exhaustion), it was shown that there is a loss in the number and functional capacity of CD8^+^ T cells during the acute phase of *P. chabaudi* malaria, which is mediated by PD-1 [9].

Several studies have shown that there is a reduction in the percentage and/or absolute number of CD8^+^ T cells in the peripheral blood during acute *P. falciparum* or *P. vivax* infection [10-14], and these reductions have been attributed to the apoptosis of these cells [15,16], the reallocation of T cells to sites of inflammation [12,17] or other factors such as the suppression of CD8^+^ T cells induced by sporozoites or infected red blood cells [18]. In regard to *P. vivax* infection, however, reports have shown that there is no significant difference in the percentage of CD8^+^ T cells during an acute malaria infection compared with that in uninfected individuals [19,20].

Considering the existing controversy regarding the role of CD8^+^ T cells during blood-stage infection, this study was conducted to quantify and evaluate the phenotypic profiling of these cells during uncomplicated symptomatic *P. vivax* malaria infection. We show that there are reduced percentages and absolute numbers of CD8^+^ naïve (CD45RA^+^), double-positive (CD45RA^+^CD45RO^+^) and memory (CD45RO^+^) T cells. Additionally, statistically significant increases in the number of CD8^+^ memory (CD45RO^+^) T cells expressing TNF-α and the number of CD8^+^ memory (CD45RO^+^) T cells expressing IL-10 were observed in *P. vivax*-infected donors*,* and a reduced absolute number of these cells expressing IFN-γ was also observed. Taken together our results suggest that *P. vivax* malaria infection reduce the number of circulating memory cells and elicit a profile of CD8^+^ T cells expressing both pro-inflammatory and anti-inflammatory cytokines, which might contribute to the clearance of the parasite without the possible harmful effect of the immunopathology.

## Methods

### Study participants and blood samples

A total of 20 subjects naturally infected with *Plasmodium vivax* (*P. vivax*-infected donors) and with uncomplicated symptomatic malaria were recruited at the Centro de Pesquisa em Medicina Tropical (Porto Velho, Rondônia–Brazil). These subjects had at least one previous malaria episode. The control group consisted of 12 healthy individuals (malaria-naïve donors) with no previous malaria exposure recruited from a non-endemic area (Belo Horizonte, Minas Gerais–Brazil). The demographic and hematological characteristics of the subjects are shown in Table 1. The parasitological diagnosis of *P. vivax* infection was conducted by thick smears technique, which was analyzed by well-trained microscopists from the Centro de Pesquisa em Medicina Tropical. The parasitemia was established in crosses and ranged from ½ + to 3+. Polymerase chain reaction (PCR) was performed to confirm *P. vivax* mono-infection using a previously described protocol [21]. Hematological parameters were measured using an automated blood cell counter (ABX Pentra 90; Horiba Diagnostics, Kyoto, Japan).

**Table 1:** **Demographic and hematological parameters of malaria-naïve donors and** ***P. vivax*** **-infected donors (mean ± SD)**

**Parameters**	**Value for group**		P value*
	**Malaria-naïve donors**	***P. vivax*** **-infected donors**	
	**(n = 11)**	**(n = 20)**	
Age (years)	29.6 (23–36)	37.1 (19–72)	
Gender			
Male	9	15	
Female	3	5	
Hemoglobin (g/dL)	14.28 ± 1.76	13.52 ± 1.21	0.138
Hematocrit (%)	44.00 ± 4.67	38.39 ± 3.16	**0.0006**
RBCs (cells/mm^3^)	4980000 ± 580000	4630000 ± 510000	0.138
WBCs (cells/mm^3^)	7958 ± 2700	5900 ± 2235	**0.014**
Lymphocytes (cells/mm^3^)	2306 ± 861.4	1583 ± 1558	**0.002**
CD8^+^	842.4 ± 320.1	352.8 ± 205.2	**<0.0001**
Monocytes (cells/mm^3^)	154.3 ± 120.8	298.7 ± 183.3	**0.018**
Granulocytes	5293 ± 2409	3686 ± 1064	**0.049**
Eosinophils (cells/mm^3^)	225.7 ± 98.75	115.2 ± 80.76	**0.002**
Platelets (cells/mm^3^)	233300 ± 78170	124100 ± 55550	**0.0001**

*Statistical differences determined by unpaired t-test. In bold significant P-values.

Peripheral venous blood was collected in heparin tubes before beginning the antimalarial treatment. These blood samples were used for cell phenotyping and to obtain plasma for the cytokine assay.

### Ethics statement

The present study was approved by the Ethics Committee of the Centro de Pesquisa em Medicina Tropical (CAAEs: 0008.0.046.000-11, 0449.0.203.000-09) and the Ethics Committee of the Universidade Federal de Minas Gerais (CAAE: 27466214.0.0000.5149). Written informed consent was obtained from each participant.

### Flow cytometric analysis

The CD8^+^ T cell analysis was conducted using four-color flow cytometry. Cell phenotyping was performed using the following monoclonal antibodies: FITC anti-human CD8 (clone HIT8a); PE anti-human IFN-γ (clone F12), TNF-α (clone MAb11), IL-10 (clone JES3-9D7), CCR7 (clone 3D12) and CD62L (clone DREG-56); PE-Cy5 anti-human CD45RO (clone UCHL1) and APC anti-human CD45RA (clone HI100). Briefly, 100 μL of fresh whole blood samples were incubated with undiluted surface monoclonal antibodies for 30 minutes at room temperature under dark conditions. Next, the erythrocytes were lysed with ammonium chloride (150 mM) for 10 minutes and washed twice in PBS-W (0.5% BSA, 0.1% sodium azide). Cells were then permeabilized with PBS-P (0.5% BSA, 0.1% sodium azide, 0.5% saponin) for 10 minutes and washed twice with PBS-W. The cells were stained with undiluted intracellular monoclonal antibodies for 30 minutes at room temperature under dark conditions, washed with PBS-W and fixed with FACS fixation solution (10 g/L paraformaldehyde, 10.2 g/L sodium cacodylate, 6.65 g/L sodium chloride) for at least 15 minutes at 4°C. Phenotypic analyses were performed using flow cytometry in a FACSCalibur flow cytometer (BD Biosciences, USA). Data were collected on 1x10^5^ lymphocytes (gated by forward and side scatter) and analyzed using Flow Jo software (Tree Star Inc., USA). The gating strategy used to characterized CD8^+^ T cells subsets was showed in Additional file 1.

### Cytokine assay

The plasma levels of IFN-γ and TNF-α were determined by enzyme-linked immunosorbent assay (ELISA) (R&D Systems) according to the manufacturer’s instructions. Biotin-labeled antibodies were used for detection and revealed with streptavidin-HRP (Amersham Biosciences, USA) and the OPD substrate system (Sigma). The colorimetric reaction was read using an automated ELISA microplate reader at 492 nm. The cytokine concentration was calculated from the standard curve using seven-parameter curve fitting software (SOFTmaxH Pro 5.3, Molecular Devices, USA), and the results were expressed in pg/mL. The limit of detection for the assay was 15.6 pg/mL for both cytokines. The IL-10 plasma level was determined using a cytometric bead array (CBA) (BD Bioscience) according to the manufacturer’s instructions. The data was collected with a FACSCan flow cytometer (BD Biosciences, USA), and the results were expressed in pg/mL. The limit of detection of the assay was 4.5 pg/mL.

### Statistical analysis

Statistical analyses were conducted using the Prism software 5.0 for Windows. Initially, the Kolmogorov-Smirnoff test was applied to verify whether the obtained data represent a normal distribution. Grubb’s test was used to verify the outliers. The unpaired t-test or Mann–Whitney test was used depending on the data distribution. A P-value < 0.05 was considered significant.

## Results

### Subsets of CD8^+^ T cells are reduced during the blood stage of *P. vivax* malaria

**Figure 1:**
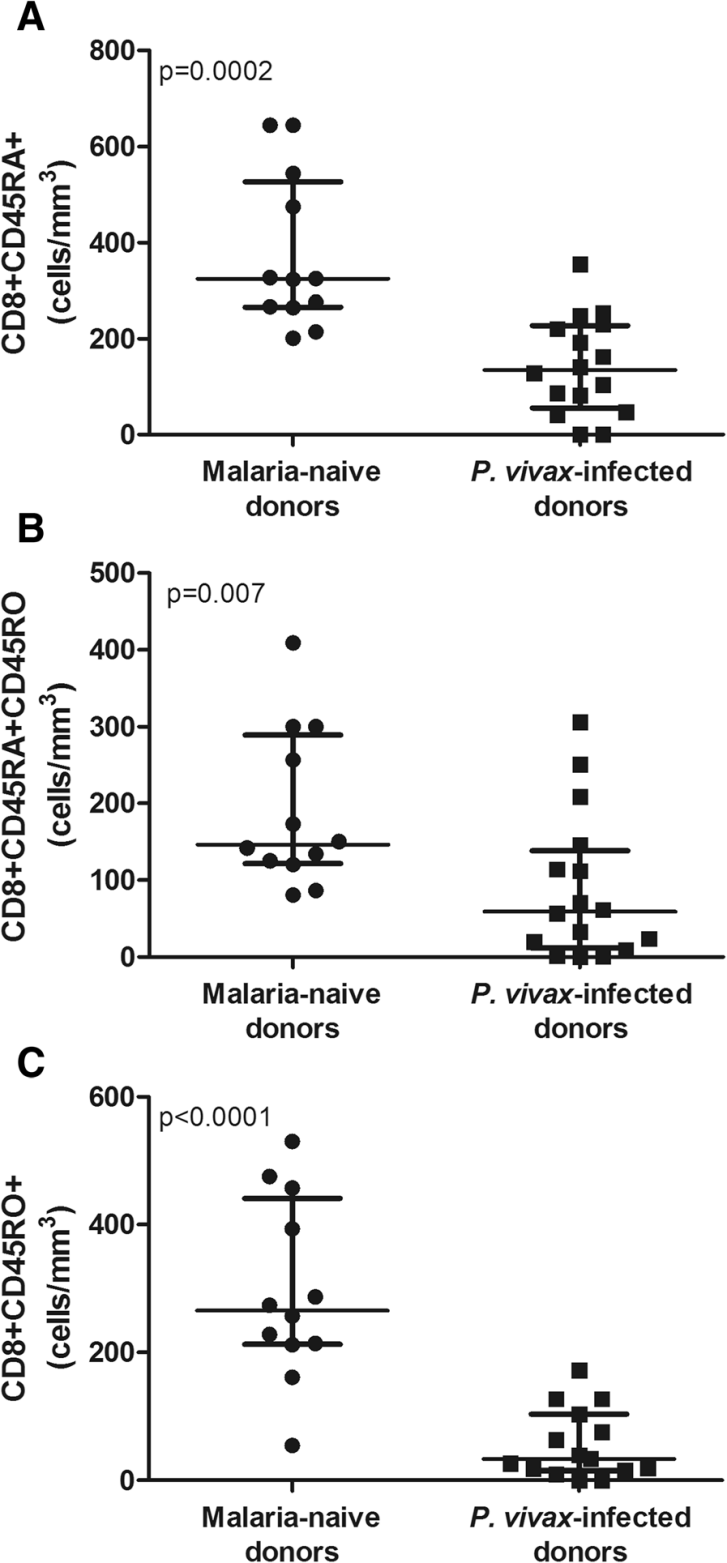
**Flow cytometric analysis of CD8** ^**+**^ **naïve, double positive and memory T cells from malaria-naïve donors (n = 12) and** ***P. vivax*** **-infected donors (n = 17). A**. CD8^+^CD45RA^+^. **B**. CD8^+^CD45RA^+^CD45RO^+^. **C**. CD8^+^CD45RO^+^. Results were expressed in absolute numbers (cells/mm^3^). Mann–Whitney test was used for comparison and the results were expressed as the median with interquartile range.

During symptomatic *P. vivax* infection, the frequency and absolute number of CD8^+^ naïve T cells (CD45RA^+^) are reduced (9.3%; median = 134.5 cells/mm^3^) compared with those in uninfected individuals (13.6%; median = 324.0 cells/mm^3^) (p = 0.0002). A similar result was found for the CD8^+^ memory T cells (CD45RO^+^) from *P. vivax*-infected donors (2.5%; median = 33.0 cells/mm^3^) compared with the malaria-naïve donors (11.8%; median = 212.5 cells/mm^3^) (p < 0.0001). An analysis of the double-positive CD8^+^CD45RA^+^CD45RO^+^ T cells also showed that the absolute number of these cells was significantly reduced in the *P. vivax*- infected donors (median = 59.5 *vs*. 146.0 cells/mm^3^) (p = 0.007) (Figure 1).

### Central memory CD8^+^ T cells are reduced during *P. vivax* infection

**Figure 2:**
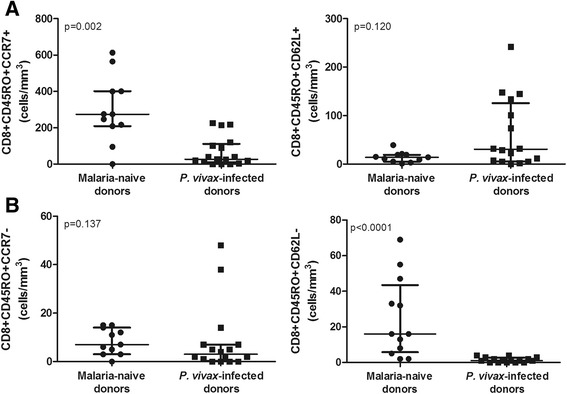
**Flow cytometric analysis of central and effector memory of CD8** ^**+**^ **T cells in malaria-naïve donors (n = 12) and** ***P. vivax*** **-infected donors (n = 17). A**. Central memory (CD8^+^CD45RO^+^CCR7^+^/CD62L^+^). **B**. Effector memory (CD8^+^CD45RO^+^CCR7^−^/CD62L^−^). Mann–Whitney test was used for comparison and the results were expressed as the median with interquartile range. P values are indicated in the graphs.

According to the expression patterns of the CCR7 and CD62L surface markers, the memory T cells can be separated into central memory (CD45RO^+^CCR7^+^/CD62L^+^) and effector memory (CD45RO^+^CCR7^−^/CD62L^−^) cells [22]. As shown in Figure 2, *P. vivax* infection reduced the number of CD8^+^CD45RO^+^CCR7^+^ compared with those in malaria-naïve donors (median = 27.0 *vs*. 274.0 cells/mm^3^), whereas there were no significant differences in CD8^+^CD45RO^+^CD62L^+^ (median = 30.5 *vs*. 14.0 cells/mm^3^) central memory T cells (p = 0.002 and p = 0.120, respectively). No difference was observed in the number of CD8^+^CD45RO^+^CCR7^−^ (p = 0.137) effector memory T cells. Despite the statistically significant result for the difference in the CD8^+^CD45RO^+^CD62L^−^ T cell number (p < 0.0001) between *P. vivax*-infected donors and malaria-naïve donors, the number of these cells was too low for this result to be meaningful (median = 1.0 *vs*. 16.0 cells/mm^3^).

### CD8^+^ naïve T cells of *P. vivax* patients present a phenotypic cytokine profile similar to that of malaria-naïve donors

**Figure 3:**
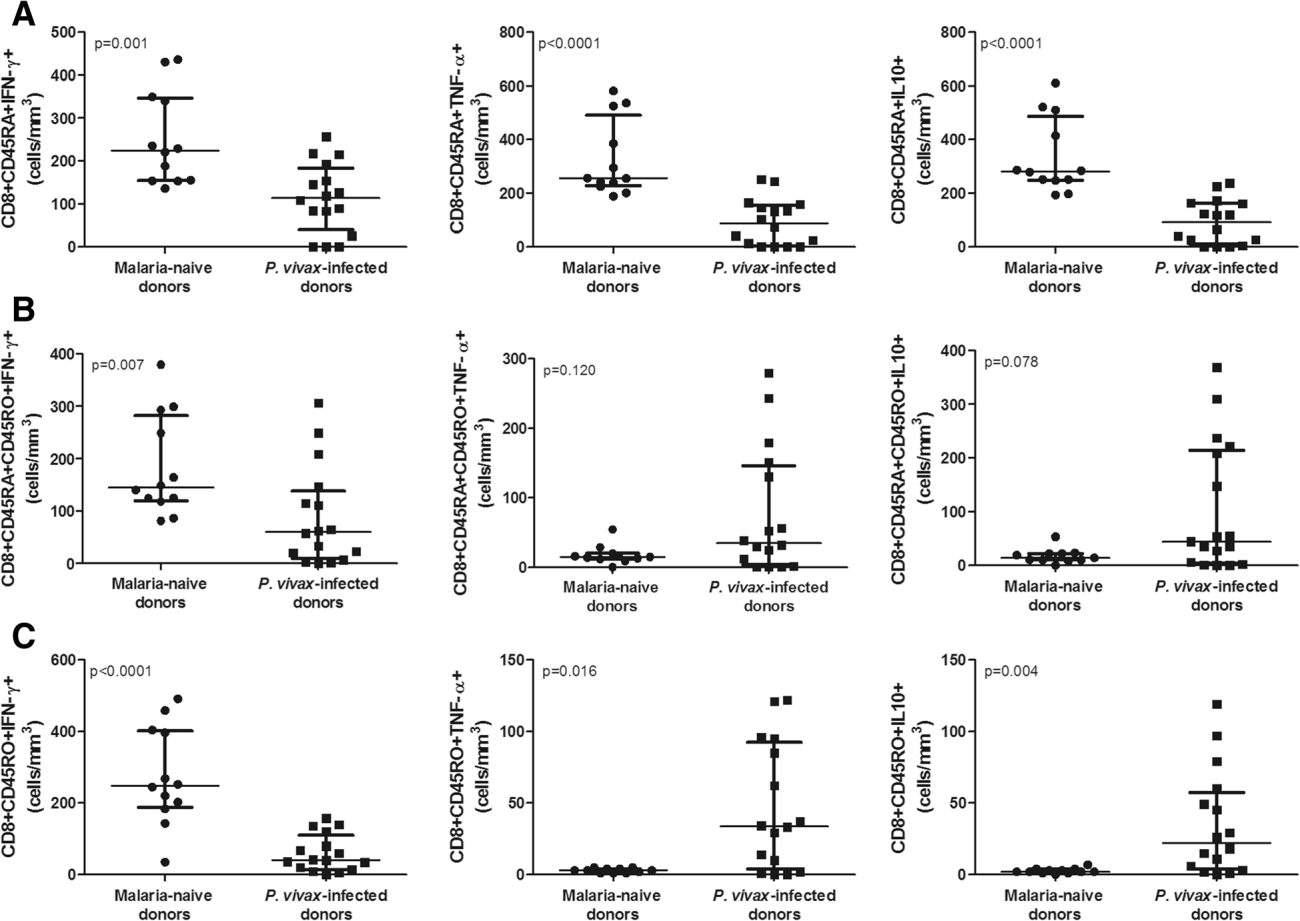
**Cytokine expression in CD8** ^**+**^ **naïve, double positive and memory T cells.** Expression of inflammatory (IFN-γ and TNF-α) and regulatory (IL-10) cytokines in malaria-naïve (n = 12) and *P. vivax*-infected donors (n = 17). The results are expressed in absolute numbers (cells/mm^3^). **A**. CD45RA^+^. **B**. CD45RA^+^CD45RO^+^. **C**. CD45RO^+^.

*P. vivax*-infected donors presented lower numbers of CD8^+^ naïve T cells IFN-γ^+^ (median = 113.0) compared with the negative controls (median = 224.5 cells/mm^3^) (p = 0.001). Similar results were observed for the CD8^+^CD45RA^+^TNF-α^+^ (median = 87.0 *vs*. 255.0 cells/mm^3^) and CD8^+^CD45RA^+^IL-10^+^ cells (median = 91.0 *vs*. 280.5 cells/mm^3^) (Figure 3A). Although the absolute numbers of these naïve T cells were lower in the *P. vivax*-infected donors, the phenotypic profiles of cytokine expression were very similar.

### CD8^+^ double-positive and memory T cells expressing TNF-α and IL-10 are enhanced during the blood stage of *P. vivax* malaria

In the *P. vivax*-infected donors, a reduced number of double-positive T cells (CD8^+^CD45RA^+^CD45RO^+^) expressing IFN-γ (p = 0.007) was observed. On the other hand, the number of these CD8^+^ T cells expressing either TNF-α^+^ or IL-10^+^ tended to increase in the *P. vivax*-infected donors (Figure 3B). Despite the reduced number of CD8^+^ memory T cells (CD8^+^CD45RO^+^) that expressed IFN-γ^+^ (p < 0.0001), the *P. vivax*-infected donors presented a significant increase in the number of memory CD8^+^ T cells expressing either TNF-α^+^ (p = 0.016) or IL-10^+^ (p = 0.004) (Figure 3C), which suggests that a different immune response occurs during blood–stage malaria infection.

### Absolute number of IL-10-producing CD8^+^ T cells correlates with the number of CD8^+^ T cells expressing IFN-γ and CD8^+^ T cells expressing TNF-α during *P. vivax* infection

**Figure 4:**
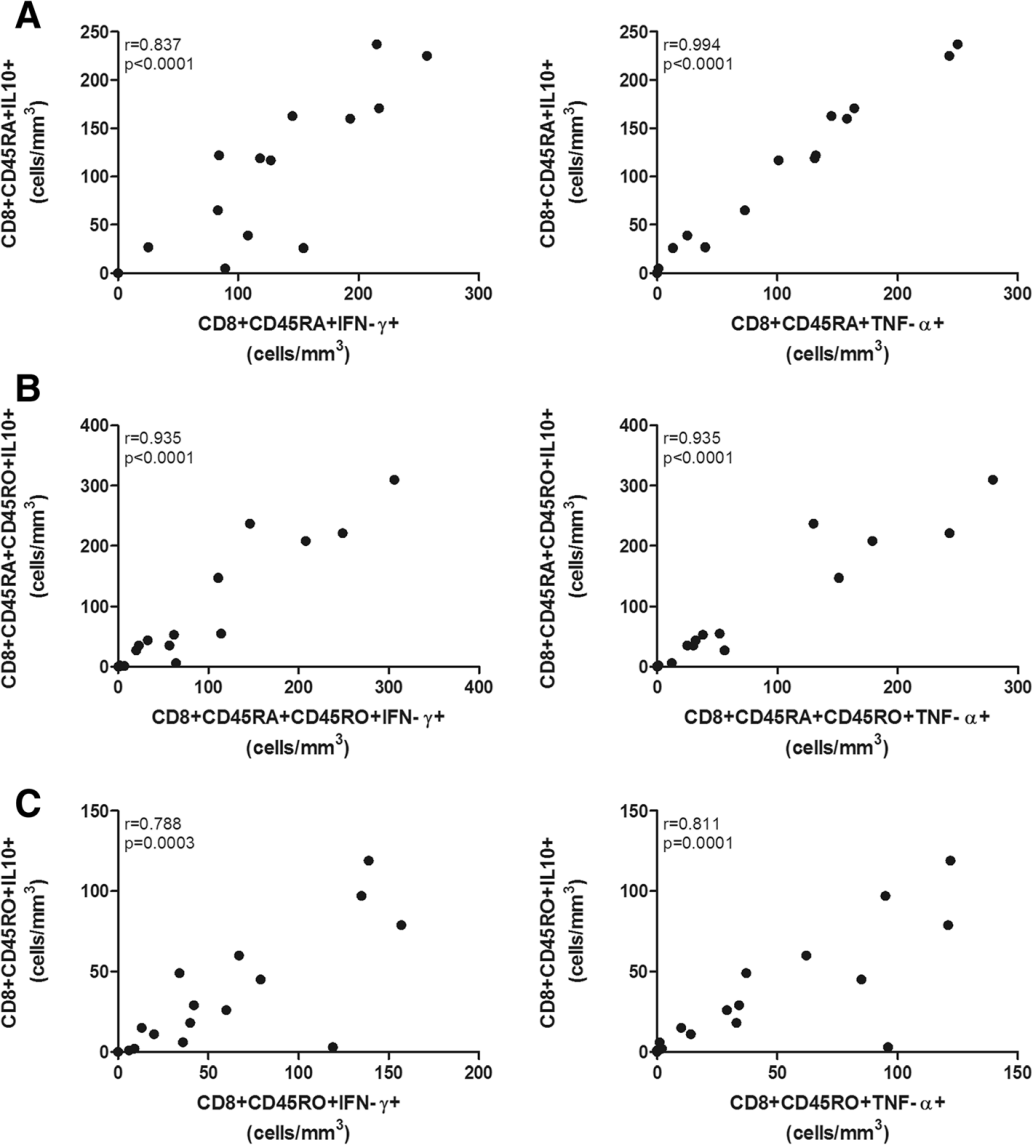
**Correlation of peripheral blood CD8** ^**+**^ **T cells expressing IL-10** ***vs*** **. IFN-γ and IL-10** ***vs*** **. TNF-α from** ***P. vivax*** **donors. A**. CD45RA^+^. **B**. CD45RA^+^CD45RO^+^. **C**. CD45RO^+^. Results are expressed in absolute numbers (cells/mm^3^). Statistical significance was determined by Spearman correlation.

The naïve, double-positive and memory CD8^+^ T cell subsets expressed pro- and anti-inflammatory cytokines, such as IFN-γ, TNF-α and IL-10. Therefore, we assessed the correlation between the numbers of CD8^+^IL-10^+^ T cells and CD8^+^ T cells with a pro-inflammatory phenotype among the different subsets of CD8^+^ T cells. Positive correlations between the absolute number of CD8^+^IL-10^+^ T cells and the numbers of CD8^+^IFN-γ^+^ (p < 0.001) and CD8^+^TNF-α^+^ T cells (p ≤ 0.0001) were found, and these correlations were independent of the subset type (naïve, double-positive and memory cells) (Figure 4A-C).

**Figure 5:**
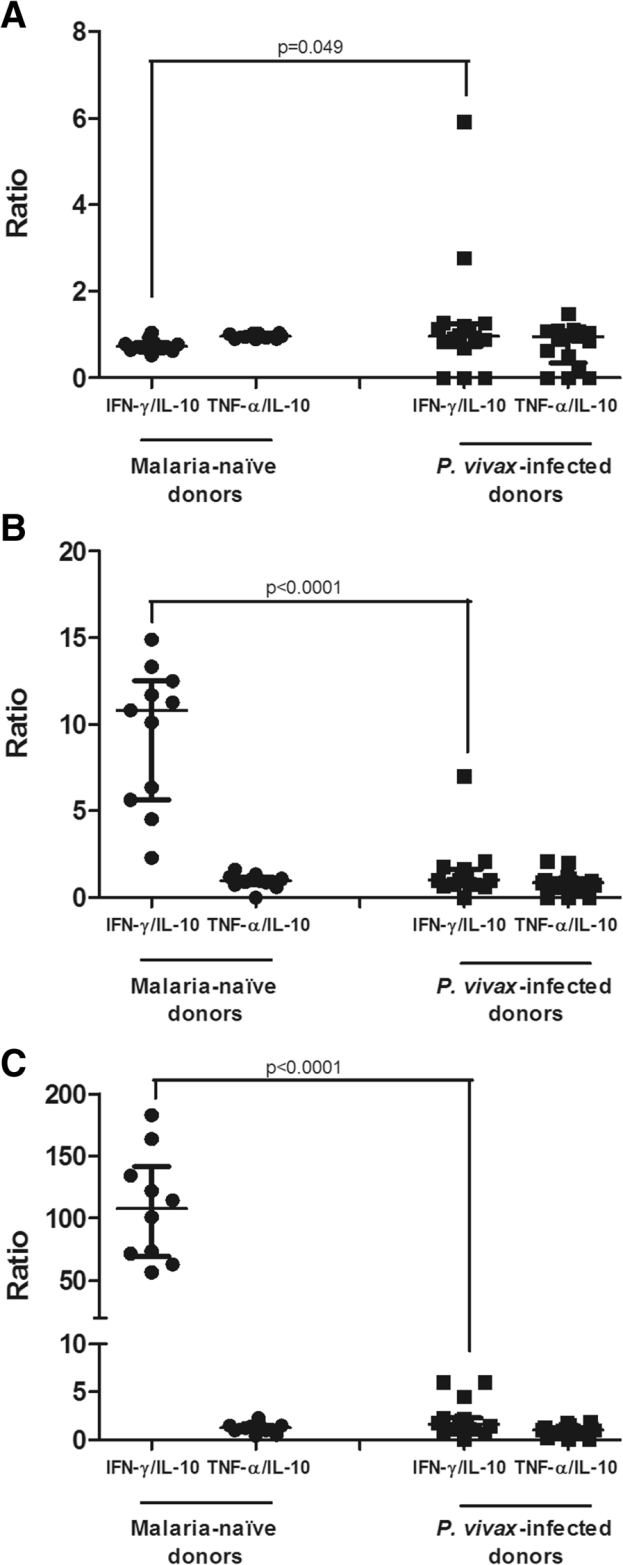
**Ratio between pro- and anti-inflammatory cytokines produced by CD8** ^**+**^ **T cells.** Results represent the ratio between pro-inflammatory (IFN-γ or TNF-α) and anti-inflammatory (IL-10) cytokine production in malaria-naïve (n = 12) and *P. vivax*-infected donors (n = 17). **A**. naïve CD45RA^+^CD8^+^ T cells. **B**. double-positive CD45RA^+^CD45RO^+^ CD8^+^ T cells. **C**. memory CD45RO^+^CD8^+^ T cells. Mann–Whitney test was used for comparison and the result was expressed as the median with interquartile range.

The analysis of ratio between production of pro-inflamatory (IFN-γ and TNF-α) and anti-inflammatory (IL-10) cytokines by T CD8^+^ cells in the different subsets are showed in Figure 5. No significant differences between *P. vivax* patients and naïve donors were detected in the ratio of TNF-α and IL-10 for all CD8^+^ T cell subsets. On the other hand, *P. vivax* malaria patients presented a significant descrease in the IFN-γ/IL-10 ratio in double-positive (CD45RA^+^CD45RO^+^) and memory (CD45RO^+^) CD8^+^ T cells when compared to control individuals, indicating a immunomodulatory profile during infection.

### Plasma levels of IFN-γ, TNF-α and IL-10 in *P. vivax*-infected donors

**Figure 6:**
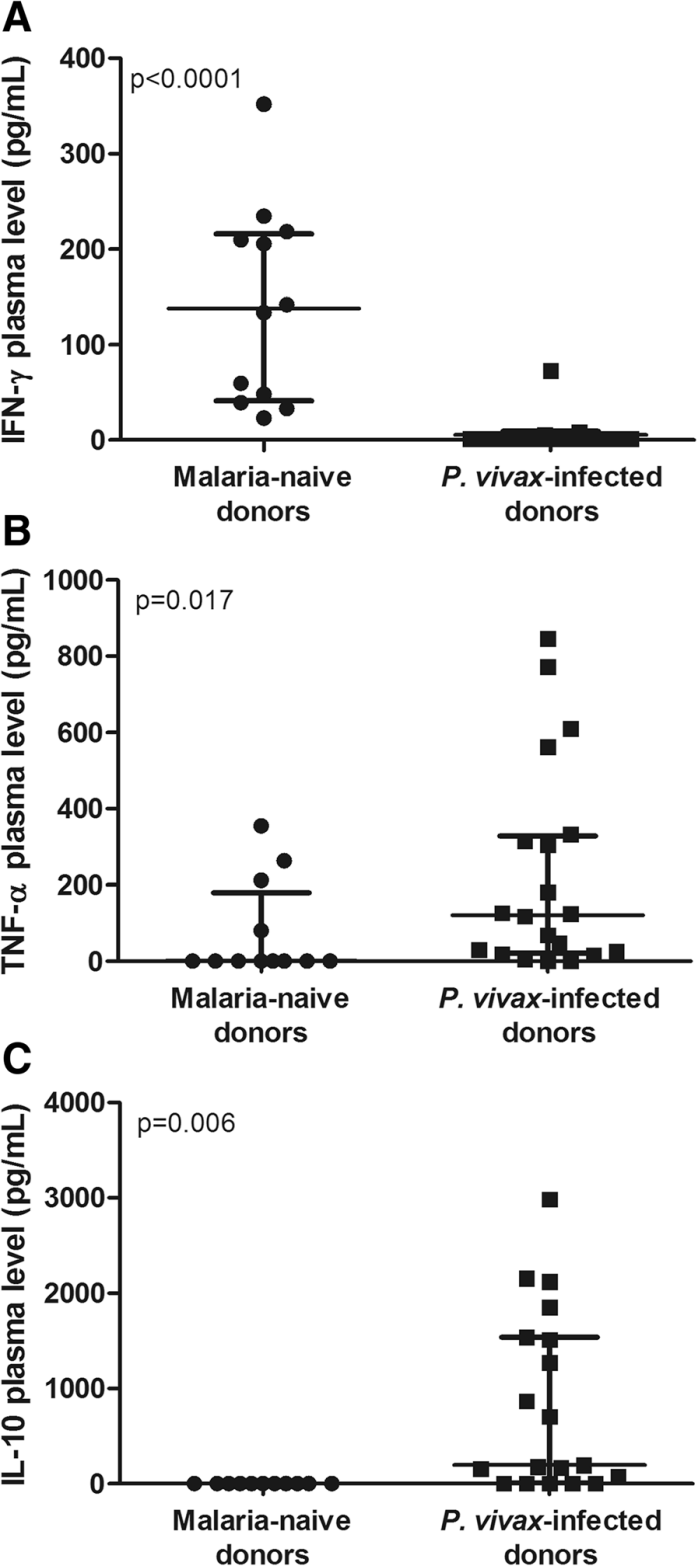
**Plasma levels of IFN-γ, TNF-α and IL-10.** Levels of circulating IFN-γ and TNF-α were determined by ELISA, IL-10 was determined by cytometric bead array (CBA) in malaria-naïve donors (n = 12) and *P. vivax*-infected donors (n = 17). **A**. IFN-γ. **B**. TNF-α. **C**. IL-10. Mann–Whitney test was used for comparison and the result was expressed as the median with interquartile range. P values are indicated in the graphs.

After evaluating the cytokine expression patterns in CD8^+^ naïve, double-positive and memory T cells, we investigated the plasma levels of IFN-γ, TNF-α and IL-10 in both donor groups. *P. vivax* infection promoted an increase in the plasma levels of TNF-α (p = 0.017) and IL-10 (p = 0.006) and a reduction in the plasma level of IFN-γ (p <0.0001) (Figure 6). These results are consistent with the phenotype of the CD8^+^ memory T cell population regarding the expression of these cytokines (Figure 3C).

## Discussion

Although studies have shown that the percentage and/or absolute number of CD8^+^ T cells are reduced during human malaria infection, the phenotypic and functional profile of these cells remain poorly characterized. Using cell surface and intracellular cytokine markers, this study aimed to identify the occurrence of different subsets of CD8^+^ T cells in individuals naturally infected with *P. vivax* during an uncomplicated symptomatic infection. For this study, blood samples obtained from patients with a *P. vivax* malarial infection and malaria-naïve donors were stained with specific monoclonal antibodies.

Our results demonstrate that during blood-stage *P. vivax* infection, there is a significant reduction in the percentages and absolute numbers of CD8^+^ naïve (CD45RA^+^) and memory (CD45RO^+^) T cells (Figure 1A and 1C). As previously discussed in the Introduction section, a number of reports have shown an overall reduction in the number of CD8^+^ T cells during an acute malaria infection in humans [10-14]. Other studies, however, have reported conflicting findings [19,20]. Several hypotheses have been proposed to explain the reduction in CD8^+^ T cell number. An interesting experimental study showed that the response of CD8^+^ T cells induced by irradiated sporozoites of *P. yoelli* in Balb/c mice is abrogated by either the simultaneous injection of nonirradiated sporozoites or the direct inoculation of blood-stage forms of the parasite [18]. Therefore, it is possible that the low number of CD8^+^ T cells observed during *P. vivax* malaria is caused by parasite-induced suppression. As an alternative possibility, Horne-Debets et al. (2013) proposed that this low number could be the result of exhaustion and a loss of CD8^+^ T cells mediated by Programmed Cell Death-1 (PD-1). Apoptosis [15,16] and the temporary reallocation of these cells to the liver and other tissues are additional alternative mechanisms that should also be considered [12,17].

Furthermore, using the CCR7 and CD62L surface markers [22], the reduced number of CD8^+^CD45RO^+^ T cells presented a central memory profile (Figure 2A). Effector memory cells migrate to tissues [22], and it is possible that the extremely low number of these cells circulating in the blood may reflect an earlier liver stage of infection. Importantly, despite the overall reduction in CD8^+^ T cells during *P. vivax* malaria, significant relative increases in the numbers of CD8^+^TNF-α^+^ and CD8^+^IL-10^+^ in the memory (CD45RO^+^) cell subsets were observed (Figure 3C), which suggests that the functional response may be different during a malaria infection. Given that double-positive (CD8^+^CD45RA^+^CD45RO^+^) T cells may represent a transition to form memory (CD8^+^CD45RO^+^) T cells, the increasing trend in the number of IL-10^+^ cells observed in *P. vivax* malaria patients may indicate the induction of a mostly immunoregulatory profile during the blood stage of this disease, as also corroborated by the analysis of the ratio between pro- and anti-inflammatory cytokines. Indeed, the number of these IL-10^+^ cells was positively correlated with the number of cells expressing IFN-γ and the number of cells expressing TNF-α. Moreover, these cell profiles are consistent with the plasma cytokine levels observed during blood-stage infection in these patients. The higher plasma levels of TNF-α and IL-10 observed in the *P. vivax*-infected donors are consistent with the results reported in other previous studies [23-25]. While the results might suggest that CD8^+^ T cells may contribute to the broad systemic changes during acute infection, it is not possible, however, to know whether these cells contribute to cytokine secretion at this stage because the number of effector cells was very low. Further studies using experimental models focusing in the depletion of specific cell populations should be carried out in order to support the importance of these cells to the broad systemic changes in the host. Conversely, the profile of the memory cells may reflect a previous exposure to the pre-erythrocytic stage antigen. From this perspective, a more immunoregulatory profile may explain the difficulty in achieving sterile immunity [3,6].

## Conclusion

Taken together, our results highlight the following two important findings: i) there is a significant reduction in specific subsets of CD8^+^ T cells, and particularly in memory cells, during the blood stage of a *P. vivax* infection; and ii) there are relatively high numbers of cells expressing IL-10 in the memory CD8^+^ T cell subsets, which positively correlates with the number of cells expressing TNF-α and the number of cells expressing IFN-γ. The results suggest that these CD8^+^ T cells co-expressing both pro-inflammatory and anti-inflammatory cytokines might contribute to the clearance of the parasite and prevent the immunopathology conferred by the excessive pro-inflammatory response. Additional studies are required to determine whether the phenotypic profiles observed represent a homeostatic immune response that benefits the host, by preventing immunopathology, or a response that is harmful due to the formation of immunomodulatory memory that would allow the persistent presence of the parasite.

## Additional file

Additional file 1: Figure S1. Gating strategy to determination of CD8^+^ T cell subsets. Representative dot plots as example of gating strategy used to characterize CD8^+^ T cells. (A) Flow cytometry pattern (FSC x SSC) of whole blood and gate on lymphocytes (B) Frequency of CD8^+^ T cells. (C) Representative dot plots showing the frequency of naïve (CD45RA^+^), double-positive (CD45RA^+^CD45RO^+^) and memory (CD45RO^+^) CD8^+^ T cells. (D) Representative dot plots showing the frequency of memory cells with expression of CCR7. Data were collected on 1×10^5^ lymphocytes (gated by forward and side scatter) and analyzed using Flow Jo software (Tree Star Inc., USA).
