# Supplementary figures and images for: Phenotypic profiling of CD8+ T cells during Plasmodium vivax blood-stage infection
Source: BMC Infect Dis. 2015 Jan 31;15:35. doi: 10.1186/s12879-015-0762-x (PMC4329216; doi:10.1186/s12879-015-0762-x)

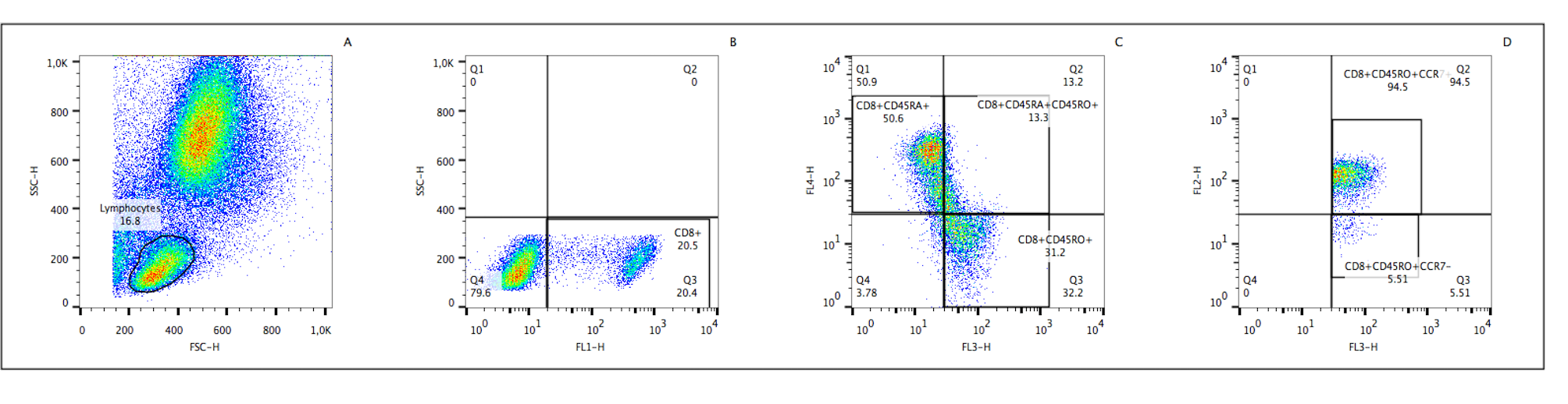

Supplement: Additional file 1: Figure S1. — Gating strategy to determination of CD8+ T cell subsets. Representative dot plots as example of gating strategy used to characterize CD8+ T cells. (A) Flow cytometry pattern (FSC x SSC) of whole blood and gate on lymphocytes (B) Frequency of CD8+ T cells. (C) Representative dot plots showing the frequency of naïve (CD45RA+), double-positive (CD45RA+CD45RO+) and memory (CD45RO+) CD8+ T cells. (D) Representative dot plots showing the frequency of memory cells with expression of CCR7. Data were collected on 1×10^5^ lymphocytes (gated by forward and side scatter) and analyzed using Flow Jo software (Tree Star Inc., USA). [file 12879_2015_762_MOESM1_ESM.tiff]
